# Cost–effectiveness thresholds: pros and cons

**DOI:** 10.2471/BLT.15.164418

**Published:** 2016-09-19

**Authors:** Melanie Y Bertram, Jeremy A Lauer, Kees De Joncheere, Tessa Edejer, Raymond Hutubessy, Marie-Paule Kieny, Suzanne R Hill

**Affiliations:** a World Health Organization, avenue Appia 20, 1211 Geneva 27, Switzerland.

## Abstract

Cost–effectiveness analysis is used to compare the costs and outcomes of alternative policy options. Each resulting cost–effectiveness ratio represents the magnitude of additional health gained per additional unit of resources spent. Cost–effectiveness thresholds allow cost–effectiveness ratios that represent good or very good value for money to be identified. In 2001, the World Health Organization’s Commission on Macroeconomics in Health suggested cost–effectiveness thresholds based on multiples of a country’s per-capita gross domestic product (GDP). In some contexts, in choosing which health interventions to fund and which not to fund, these thresholds have been used as decision rules. However, experience with the use of such GDP-based thresholds in decision-making processes at country level shows them to lack country specificity and this – in addition to uncertainty in the modelled cost–effectiveness ratios – can lead to the wrong decision on how to spend health-care resources. Cost–effectiveness information should be used alongside other considerations – e.g. budget impact and feasibility considerations – in a transparent decision-making process, rather than in isolation based on a single threshold value. Although cost–effectiveness ratios are undoubtedly informative in assessing value for money, countries should be encouraged to develop a context-specific process for decision-making that is supported by legislation, has stakeholder buy-in, for example the involvement of civil society organizations and patient groups, and is transparent, consistent and fair.

## Résumé

Les analyses de rentabilité permettent de comparer les coûts et les résultats de différentes options politiques. Chaque ratio coût-efficacité qui en découle indique l'importance des avantages supplémentaires pour la santé par unité supplémentaire de ressources dépensée. Les seuils de rentabilité permettent de déterminer les ratios coût-efficacité qui représentent une bonne ou une très bonne rentabilité. En 2001, la Commission macroéconomie et santé de l'Organisation mondiale de la Santé a suggéré des seuils de rentabilité définis d'après des multiples du produit intérieur brut (PIB) par habitant d'un pays. Dans certains pays, ces seuils ont servi de règles pour décider quelles interventions financer ou non. Cependant, l'expérience d'utilisation de ces seuils fondés sur le PIB dans les processus décisionnels des pays montre qu'ils ne tiennent pas compte des spécificités des pays; cela, ajouté à une certaine incertitude concernant la modélisation des ratios coût-efficacité, peut entraîner la prise de mauvaises décisions quant à l'utilisation des ressources sanitaires. Les informations sur la rentabilité des interventions devraient être prises en compte parallèlement à d'autres considérations, comme l'impact budgétaire et la faisabilité, dans le cadre d'un processus décisionnel transparent et non de façon isolée sur la base d'une seule valeur seuil. Bien que le caractère informatif des ratios coût-efficacité soit indéniable lorsqu'il s'agit d'évaluer la rentabilité des interventions, les pays devraient être encouragés à développer un processus de prise de décision spécifique au contexte, qui soit encadré par la législation et qui ait l'adhésion des parties intéressées, avec par exemple l'implication d'organisations de la société civile et de groupes de patients, et qui soit transparent, cohérent et équitable.

## Resumen

El análisis de rentabilidad se utiliza para comparar los costes y resultados de opciones políticas alternativas. Cada relación de rentabilidad resultante representa la magnitud de sanidad adicional obtenida por unidad adicional de recursos utilizados. Los umbrales de rentabilidad permiten la identificación de las relaciones de rentabilidad que representan un valor bueno o muy bueno del capital. En 2001, los umbrales de rentabilidad propuestos por la Comisión sobre Macroeconomía y Salud de la Organización Mundial de la Salud se basaron en múltiplos del producto interior bruto (PIB) per cápita de un país. En algunos contextos, se han utilizado estos umbrales para decidir qué intervenciones sanitarias financiar y cuáles no. No obstante, la experiencia con el uso de dichos umbrales basados en el PIB en los procesos de toma de decisiones a nivel nacional muestra la ausencia de especificidad según el país. Esto, además de la incertidumbre de las relaciones de rentabilidad modelo, puede dar lugar a una toma de decisiones equivocada sobre cómo emplear los recursos sanitarios. La información relativa a la rentabilidad debería utilizarse teniendo en cuenta otros factores (por ejemplo, el impacto presupuestario y aspectos de viabilidad) en un proceso transparente de toma de decisiones, en lugar de únicamente teniendo como referencia un solo valor del umbral. A pesar de que las relaciones de rentabilidad son indudablemente esclarecedoras a la hora de evaluar el valor del capital, es necesario fomentar que los países desarrollen un proceso específico del contexto apoyado por la legislación para tomar decisiones, como, por ejemplo, si las partes interesadas han aceptado la implicación de las organizaciones de la sociedad civil y grupos de pacientes y si es transparente, coherente y justa.

## ملخص

تُستخدم تحاليل فعالية التكلفة للمقارنة بين التكاليف والحصائل الناتجة عن خيارات السياسات البديلة. تمثل كل نسبة من نسب فعالية التكلفة الناتجة حجم المستوى الصحي الإضافي المكتسب من استهلاك وحدة إضافية من الموارد. وتتيح القيم الحدية لفعالية التكلفة تحديد نسب فعالية التكلفة التي تمثل قيمة جيدة أو قيمة جيدة جدًا مقابل المال. وكانت اللجنة التابعة لمنظمة الصحة العالمية والمعنية بالاقتصاد الكلي في مجال الصحة قد اقترحت في عام 2001 إنشاء قيم حدية لفعالية التكلفة بناءً على مضاعفات نصيب الفرد من إجمالي الناتج المحلي (GDP) في البلاد. وفي بعض الظروف، تم استخدام هذه القيم الحدية باعتبارها قواعد لاتخاذ القرار باختيار أي التدخلات الصحية ينبغي تمويلها أو عدم تمويلها. إلا أن واقع تجربة استخدام هذه القيم الحدية المستندة إلى إجمالي الناتج المحلي في عمليات اتخاذ القرار على المستوى القطري يبين أنها تفتقر إلى التخصصية في البلدان. هذا بالإضافة إلى عدم اليقين بنسب فعالية التكلفة النموذجية يمكن أن يؤدي إلى اتخاذ قرار خاطئ بشأن طريقة استهلاك موارد الرعاية الصحية. ينبغي استخدام المعلومات المتعلقة بفعالية التكلفة، إلى جانب اعتبارات أخرى، على سبيل المثال اعتبارات تأثير الميزانية ومدى الجدوى في عملية اتخاذ قرارات تتسم بالشفافية عوضًا عن اتخاذ قرارات منعزلة مستندة إلى قيمة حدية فردية. على الرغم من أن نسب فعالية التكلفة مفيدة بلا شك في تقدير القيمة مقابل المال، ينبغي تشجيع البلدان على إعداد عمليات متناسبة مع الظروف لاتخاذ القرارات المدعومة بالتشريعات، في حال قررت الجهات المعنية، على سبيل المثال إشراك منظمات المجتمع المدني ومجموعات المرضى، وتتسم هذه القرارات بأنها شفافة ومتسقة ونزيهة.

## 摘要

成本效益分析用于比较备选政策选项的成本及效果。 每个生成的成本效益比代表着每增加一个单位的资源投入所获得的额外健康收益的数量。 成本效益阈值确定代表物有所值或物超所值的成本效益比。 2001 年,世界卫生组织宏观经济与卫生健康委员会提出基于国家人均国内生产总值 (GDP) 倍数的成本效益阈值。 某些情况下,在选择资助和不资助哪些健康干预项目方面,这些阈值已被用作决策规则。 然而,在将这种基于 GDP 的阈值用于国家级决策过程中的经验中,显示出这些阈值缺乏国家针对性,并且——除了实现模型成本效益比的不确定性之外——这将可能导致在如何使用卫生保健资源上做出错误决策。 成本效益信息应与其他因素——如:预算影响和可行性因素——一起用于透明的决策过程当中,而不是孤立地基于一个单阈值。 尽管成本效益比在评估性价比方面无疑提供了有用信息,但应鼓励各国建立受法律支持、利益相关者认可(如:民间社会组织与患者群体的参与)、透明、一致且公正的基于具体国情的决策制定过程。

## Резюме

Анализ экономической эффективности применяется для сравнения затрат и результатов различных вариантов одной и той же стратегии. Во всех случаях соотношение затрат и эффективности дает представление о том, как будет происходить улучшение здоровья на единицу затраченных ресурсов. Пороговое значение экономической эффективности позволяет выявить такое соотношение затрат и эффективности, которое отвечает получению хороших или очень хороших результатов относительно затраченных средств. В 2001 году комиссия Всемирной организации здравоохранения по макроэкономике в здравоохранении порекомендовала пороговые значения экономической эффективности, основанные на параметрах валового внутреннего продукта на душу населения (ВВП) стран. В некоторых ситуациях эти пороговые значения использовались для принятия решения о том, какие меры по здравоохранению будут получать финансирование, а какие — нет. Однако опыт применения пороговых значений, основанных на величине ВВП стран, показал, что при этом не учитывается их специфика, и это в сочетании с неопределенностью в описании моделируемых затрат может способствовать принятию неверных решений о распределении средств на охрану здоровья. Информацию об экономической эффективности следует использовать с учетом других факторов, например влияния на бюджет и возможности осуществления, при этом процесс принятия решений должен быть прозрачным и выполняться не только на основе одного порогового показателя. Хотя соотношение затрат и эффективности, без сомнения, позволяет определенным образом оценить пользу от затраченных средств, следует поощрять страны на развитие прозрачным, честным и единообразным способом, учитывая их собственную ситуацию при разработке процесса принятия решений, который поддерживается законодательством и имеет заинтересованных акционеров, например гражданских ассоциаций или групп пациентов.

## What are cost–effectiveness thresholds?

The main results of a cost–effectiveness analysis – in which the costs and outcomes of alternative policy options are compared – are cost–effectiveness ratios. In the field of health, a cost–effectiveness ratio usually represents the amount of additional health gained for each additional unit of resources spent. The makers of health policy initially used cost–effectiveness analyses for priority setting, in their attempts to ensure that the greatest possible health benefits were achieved given the available budget. Many countries currently use cost–effectiveness analyses and the resultant cost–effectiveness ratios to guide their decisions on resource allocation and to compare the efficiencies of alternative health interventions.

A cost–effectiveness threshold is generally set so that the interventions that appear to be relatively good or very good value for money can be identified. There are several types of threshold. In health-related analyses, a willingness-to-pay threshold represents an estimate of what a consumer of health care might be prepared to pay for the health benefit – given other competing demands on that consumer’s resources. There are also supply-side thresholds that take resource allocation into account – e.g. estimates of the health foregone because an insurance company or other provider spends some of its available budget on a new intervention and is therefore forced to reduce its funding of older interventions.

In considering the choice of the type of cost–effectiveness threshold to use, the concept of opportunity cost may be the one most relevant to providers who are primarily concerned with using the available resources to improve health. In considering the implementation of a new intervention, decision-makers need estimates of both the health that might be gained elsewhere through the alternative use of the resources needed for the new intervention and the health that is likely to be lost if the new intervention is not used.

Recent claims about the misapplication of cost–effectiveness thresholds^1^ are well founded. However, we feel that the implication that the World Health Organization’s (WHO’s) Commission on Macroeconomics and Health’s cost–effectiveness thresholds are intended to be used as the explicit criteria for health decisions at national level – ignoring all other policy-relevant evidence – is incorrect.

## Thresholds based on gross domestic product

The most commonly cited cost–effectiveness thresholds are those based upon a country’s per-capita gross domestic product (GDP) and the Commission on Macroeconomics and Health’s corresponding estimate of the economic value of a year of healthy life.^2^ As ill health has a negative economic impact, investments in health can contribute to economic development. The commission, in trying to encourage investment in health, has suggested that all countries should map out a path to universal access to essential health services, increase domestic financing for health and include economic considerations in their attempts to identify health priorities.^2^ The commission also suggested that it was reasonable to spend the estimated value of a year of healthy life, per capita, on an intervention that led to a mean of at least one additional year of healthy life per capita.^2^

The commission’s GDP-related cost–effectiveness thresholds were based on assumptions about leisure time, non-health consumption, longevity and health-related quality of life. They can be compared to measures – e.g. the so-called value of a statistical life – that are based on individuals’ actual choices^3^ (DT Jamison, personal communication, 2015) and represent an estimate of an individual’s willingness to pay to extend their healthy life by one year. There has been criticism of the commission’s focus on GDP-based thresholds, since “people value life in dimensions that extend beyond income”.^4^ However, the cost thresholds published by the commission in 2001^2^ are similar to the more detailed – and, perhaps, more reliable – estimates published over a decade later.^5^

In 2005, authors writing on behalf of WHO’s Choosing Interventions that are Cost–Effective project (WHO-CHOICE) suggested that “interventions that avert one DALY [disability-adjusted life-year] for less than average per capita income for a given country or region are considered very cost–effective; interventions that cost less than three times average per capita income per DALY averted are still considered cost–effective; and those that exceed this level are considered not cost–effective”.^6^ Although they may indicate that an intervention is cost–effective or very cost–effective, none of these thresholds should be used, alone, as a decision rule for funding or as a measure of affordability. They are simply an indication that, in a given setting, an intervention may represent poor, good or very good value for money.

As used by WHO-CHOICE, the Commission on Macroeconomics and Health’s GDP-based thresholds were only intended to be generic global norms. For example, the list of interventions given in Appendix 3 of the WHO’s Global Action Plan for the Prevention and Control of Noncommunicable Diseases 2013–2020^7^ – i.e. the list of interventions sometimes referred to as the best buys – represents a menu of medical and public health interventions to consider in a range of settings. Although this list was partly based on value for money – in terms of GDP-based cost–effectiveness thresholds – it was also based on affordability, feasibility and other criteria. In a similar manner, in work carried out on behalf of WHO-CHOICE, GDP-based thresholds were used to categorize interventions as cost–effective or very cost–effective but the intention was only to guide policy-makers on value for money.^8^ It was always assumed and intended that other considerations relevant to local settings would be used in decision-making.

## Interpreting WHO-CHOICE’s results

The main objective of WHO-CHOICE is to assist with priority setting across an entire benefits package – and, ultimately, achieve universal health coverage. Other related programmes for priority setting – e.g. the SMART vaccine project^9^ – use the results of cost–effectiveness analysis only to make incremental or marginal decisions about the addition of single interventions to an existing benefits package. Where the primary goal of a health system is the optimization of population health, it can be important to use an approach such as that followed by WHO-CHOICE – and its generalized cost–effectiveness analysis – to decide which set of interventions, out of a larger group of feasible options, offer the best value for money. The addition of single interventions one at a time, based on incremental analyses, may not result in the optimal use of resources. However, given that many systems already have an existing package of interventions, in some settings there is clearly still a role for incremental analysis.

## Misuse of thresholds

Many factors influence the results of cost–effectiveness analyses – e.g. the data used to estimate costs and effects, the choice of comparator and whether or not subgroups of the target population are analysed. Variations in the inputs can have substantial effects on the estimate of a cost–effectiveness ratio. If the analyses do not reflect the policy context accurately, overreliance on cost–effectiveness ratios and a fixed cost–effectiveness threshold, to guide decision-making, may result in the wrong decisions being made.

At a technical level, it is important to note that cost–effectiveness ratios derived from economic modelling are simply estimates – generally based on several assumptions – produced to indicate the potential value for money of one or more interventions. The construction of economic models is prone to problems and errors,^10^^–^^15^ but such models can still be a valuable input for decision-making if well-constructed and validated. However, even well-constructed models can produce a range of estimates depending on the assumptions adopted and the formulation of the policy question being evaluated. Use of a rigid cost–effectiveness threshold to determine funding decisions may simply encourage the interested parties to tailor their estimates so that they trigger funding.

Even if estimated accurately, generic GDP-based cost–effectiveness ratios – or other estimates of willingness to pay – do not provide information on affordability, budget impact or the feasibility of implementation. In Peru, a contextualised WHO-CHOICE analysis of breast cancer treatments concluded that addition of trastuzumab to a package of interventions would be cost–effective – i.e. cost less than three times the per-capita GDP per DALY averted.^16^ However, the costs of adding trastuzumab would exceed Peru’s entire budget for breast cancer treatment.^16^

Similarly, several analyses have concluded that sofosbuvir is a cost–effective treatment option for some subgroups of patients with hepatitis C.^17^^–^^19^ For example, using a cost– effectiveness threshold of 100 000 United States dollars per DALY averted, it was estimated that 83% of hepatitis C patients in the United States of America would be eligible for treatment with the drug.^17^ However, treatment of all the eligible patients would require a 4% increase in national pharmaceutical spending. Such an increase is probably unaffordable and more cost–effective interventions would probably be crowded out if sofosbuvir were to be offered on such a large scale.

In the detection of tuberculosis, the use of GeneXpert (Cepheid, Sunnyvale, United States of America) – a molecular test for the deoxyribonucleic acid of *Mycobacterium tuberculosis* – is considered to be a cost–effective intervention that has already been implemented in South Africa.^20^ Widespread use of the test not only has high initial costs – in terms of laboratory space, GeneXpert machines and staff training – but also depends on a consistent electrical supply.^21^ In the absence of basic amenities such as regular electricity supply, any GeneXpert machines are likely to remain underused and unable to achieve their modelled levels of efficiency and cost–effectiveness.

## From evidence to decision-making

The use of cost–effectiveness ratios in decision-making remains an area without consensus.^15^ Our view is that a fixed cost–effectiveness threshold should never be used as a stand-alone criterion for decision-making. Above all, the indiscriminate sole use of the most common threshold – of three times the per-capita GDP per DALY averted – in national funding decisions or for setting the price or reimbursement value of a new drug or other intervention must be avoided. WHO-CHOICE has never recommended this practice, which would be a distortion of the intention and meaning of the GDP-based thresholds proposed by the Commission on Macroeconomics and Health.

If a single fixed cost–effectiveness threshold is not to be used – at least, not alone – what are the alternatives? In the development of clinical guidelines, evidence-to-decision frameworks have been developed to guide decision-making.^22^^,^^23^ Explicit guidance on the inclusion of fairness in the decision-making needed to achieve universal health coverage has been published.^24^ Multi-criteria decision analysis frameworks have also been suggested.^25^

Based on our experience, we believe that countries should consider establishing a context-specific process for decision-making that is supported by legislation, has stakeholder buy-in and is consistent, fair and transparent. While cost–effectiveness ratios are undoubtedly informative in assessing value for money – from either the supply or demand side – they also need to be considered alongside affordability, budget impact, fairness, feasibility and any other criteria considered important in the local context. The Norwegian Committee on Priority Setting has proposed the use of three criteria – i.e. health benefit, health loss and resources – and suggested differentiating thresholds across the different categories of potential health loss.^26^

Decision-makers need to have sufficient confidence in the quality and reliability of cost–effectiveness estimates, which, in turn, requires sufficient local capacity for the appraisal of economic models and their outputs. In health systems that have these components in place, a more meaningful local and explicit cost–effectiveness threshold might eventually emerge (Box 1). To ensure better health outcomes and optimal value for money, decision-makers need to use all the relevant data and estimates wisely.

Box 1 **Experiences with the use of explicit cost–effectiveness thresholds**

**Australia**

A retrospective analysis of the recommendations of the Pharmaceutical Benefits Advisory Committee found that the implied threshold for a positive recommendation was 46 400 Australian dollars – i.e. 1.35 times the per-capita gross domestic product (GDP) in 1999 – per quality-adjusted life-year (QALY) gained.^27^ However, it was noted that there was, in fact, no fixed threshold and that other aspects of the related evidence – e.g. confidence in the clinical data – appeared to have been just as important to the committee as estimated cost–effectiveness ratios.^27^^,^^28^ The committee has experts who review all submissions and has the legislative mandate to provide advice on reimbursement prices.

**Poland**

In 2012, for its decisions on reimbursing the costs of new pharmaceuticals, Poland legislated a cost–effectiveness threshold of three times the per-capita gross GDP per QALY gained.^29^ Manufacturers who submit applications for reimbursement of the costs of new products are required to provide fully-functional models that allow the evaluation of all the input parameters. Although the impact of the threshold is not yet clear, the prices paid in Poland for certain products appear to be higher than the mean values for the European Union.^30^

**Thailand**

In 2007, the subcommittee responsible for the development of Thailand’s national list of essential medicines set a threshold of 100 000 Thai baht – i.e. 0.8 of the per-capita GDP – per QALY gained.^31^ This threshold, which applies specifically to medicines included on the essential medicines list, has been a particularly powerful tool in price negotiations. For example, it has resulted in price decreases, in Thailand, of 72% for tenofovir and 69% for oxaliplatin.^32^ Health technology assessments are commissioned through the Health Intervention and Technology Assessment Programme and made independently of any pharmaceutical company. Decisions on the benefit package are made by the National Health Assembly, using societal values, and cost–effectiveness thresholds are therefore not the only aspect taken into consideration.^33^

**United Kingdom of Great Britain and Northern Ireland**

Since at least 2000, the United Kingdom’s National Institute for Health and Care Excellence has used an explicit cost–effectiveness threshold of between 20 000 and 30 000 pounds sterling (£) – i.e. 1.18 and 1.76 times the per-capita GDP in 2000, respectively, but only 0.70 and 1.04 times the corresponding product for 2015, respectively – per QALY gained. If the incremental cost–effectiveness ratio for a new technology falls below £20 000 per quality-adjusted life-year gained, that technology is generally recommended for purchase by the national health system. Technologies that appear less cost–effective may still be recommended if they are for end-of-life care or for diseases associated with short life expectancies that would be extended by the technology. However, when some cancer drugs were consistently found to have cost–effectiveness ratios of more than £30 000 per QALY gained – and were therefore rejected by the National Institute for Health and Care Excellence – an alternative funding mechanism was established. The National Institute’s effective cost–effectiveness threshold – reflecting the likely impact of expenditure on both mortality and morbidity – has been estimated to be £12 936 per QALY gained. This relatively low value probably reflects the displacement of more cost–effective activities by new approvals.^34^^,^^35^
